# Casticin induces apoptosis and G0/G1 cell cycle arrest in gallbladder cancer cells

**DOI:** 10.1186/s12935-016-0377-3

**Published:** 2017-01-05

**Authors:** Xiao-ling Song, Yun-jiao Zhang, Xue-feng Wang, Wen-jie Zhang, Zheng Wang, Fei Zhang, Yi-jian Zhang, Jian-hua Lu, Jia-wei Mei, Yun-ping Hu, Lei Chen, Huai-feng Li, Yuan-yuan Ye, Ying-bin Liu, Jun Gu

**Affiliations:** 1 Department of General Surgery and Laboratory of General Surgery, Xinhua Hospital Affiliated to Shanghai Jiao Tong University School of Medicine, Shanghai, People’s Republic of China; 2 Department of Cardio-Thoracic Surgery, Xinhua Hospital Affiliated to Shanghai Jiao Tong University School of Medicine, Shanghai, People’s Republic of China; 3 Institute of Biliary Tract Disease, Shanghai Jiao Tong University School of Medicine, Shanghai, People’s Republic of China

**Keywords:** Casticin, Gallbladder cancer, Akt signaling pathway, G0/G1 arrest, Apoptosis

## Abstract

**Background:**

Casticin, the flavonoid extracted from *Vitex rotundifolia* L, exerts various biological effects, including anti-inflammatory and anti-cancer activity. The aim of this study is to investigate the effects and mechanisms of casticin in human gallbladder cancer cells.

**Methods:**

Human NOZ and SGC996 cells were used to perform the experiments. CCK-8 assay and colony formation assay were performed to evaluate cell viability. Cell cycle analyses and annexin V/PI staining assay for apoptosis were measured using flow cytometry. Western blot analysis was used to evaluate the changes in protein expression, and the effect of casticin treatment in vivo was experimented with xenografted tumors.

**Results:**

In this study, we found that casticin significantly inhibited gallbladder cancer cell proliferation in a dose- and time-dependent manner. Casticin also induced G0/G1 arrest and mitochondrial-related apoptosis by upregulating Bax, cleaved caspase-3, cleaved caspase-9 and cleaved poly ADP-ribose polymerase expression, and by downregulating Bcl-2 expression. Moreover, casticin induced cycle arrest and apoptosis by upregulating p27 and downregulating cyclinD1/cyclin-dependent kinase4 and phosphorylated protein kinase B. In vivo, casticin inhibited tumor growth.

**Conclusion:**

Casticin induces G0/G1 arrest and apoptosis in gallbladder cancer, suggesting that casticin might represent a novel and effective agent against gallbladder cancer.

## Background

Gallbladder cancer (GBC) is the most common malignant and fatal tumor of the biliary tract [1]. Diagnostic and prognostic markers for this malignancy have not been extensively studied, and, due to lack of conspicuous symptoms and physical signs, the majority of patients are diagnosed at an advanced and incurable stage [2, 3]. Moreover, GBC is resistant to chemotherapy or radiotherapy, surgical resection is the only potentially effective treatment for GBC [4, 5]. As a result, the overall 5-year survival rate of GBC is less than 5% [5, 6]. Therefore, the development of novel and effective agents for GBC treatment remains a significant challenge.

Flavonoids, which are plentiful in components of human diets, such as fruits and vegetables, exhibit extensive biological effects, including anti-cancer, anti-inflammatory, anti-oxidant and anti-viral activities [7]. Casticin, the flavonoid extracted from *Vitex rotundifolia* L, exerts anti-inflammatory and anti-cancer activities. Casticin has been commonly used as an anti-inflammatory agent for thousands of years in traditional Chinese medicine [8]. In addition, resent studies has demonstrated that casticin can alleviate smoke-induced acute lung inflammation [9]. In recent years, researchers have focused their attention on the anti-cancer effects of casticin against lung cancer, cervical cancer, hepatocellular carcinoma, colon cancer and gastric cancer [10–14]. However, the effects and mechanisms of casticin on human GBC cells have yet to be characterized.

In this study, we explored the anti-cancer effect of casticin on GBC cells and investigated the potential mechanisms mediating these effects. We found that casticin induced G0/G1 arrest and apoptosis in gallbladder cancer, suggesting that casticin might represent a novel and effective agent against gallbladder cancer.

## Methods

### Reagents and drugs

**Fig. 1:**
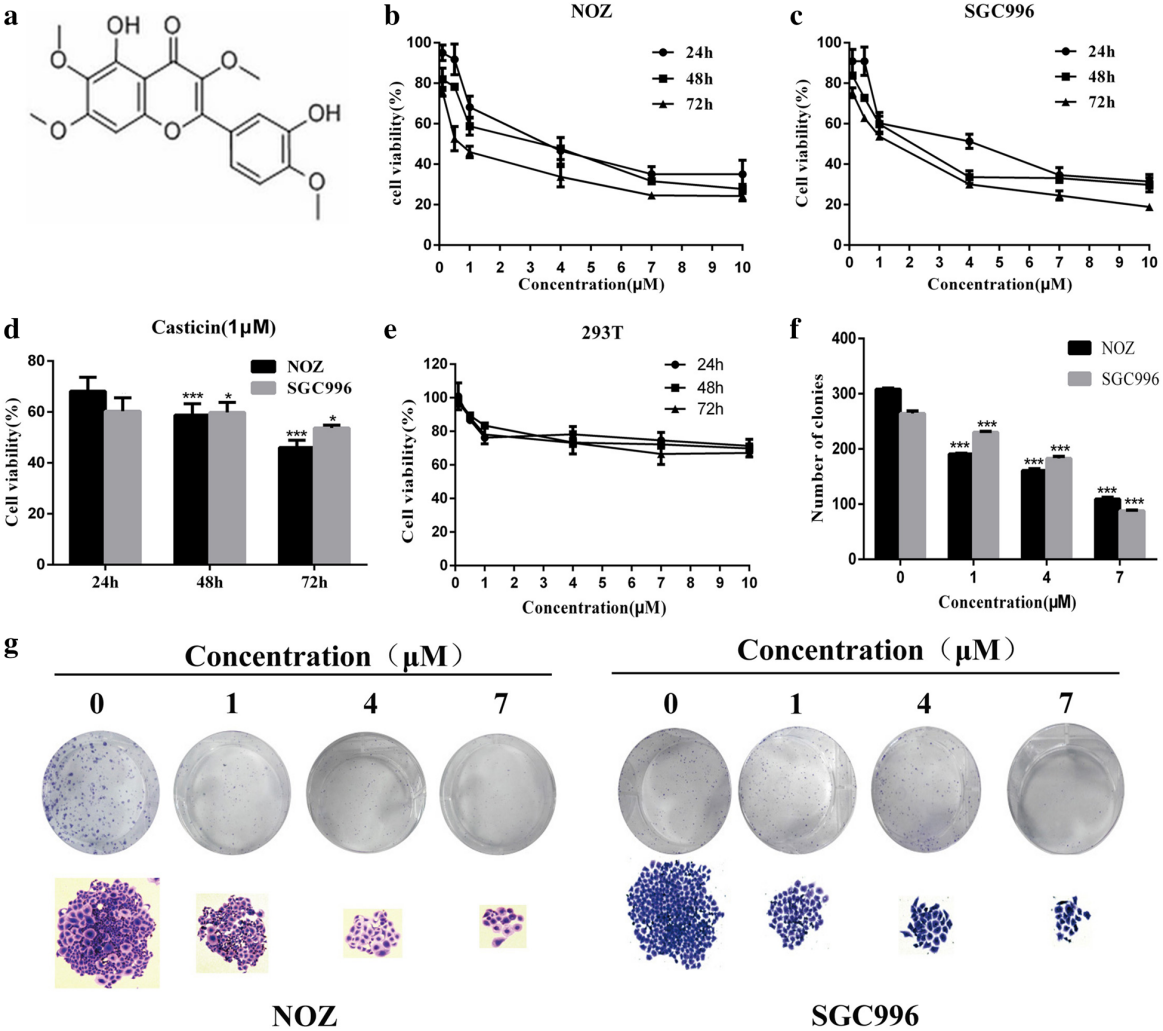
Casticin inhibits the proliferation and viability of NOZ and SGC996 cells. **a** The chemical structure of casticin. **b**, **c** NOZ, SGC996 and 293T cells were treated with various concentrations of casticin (0, 0.1, 0.5, 1, 4, 7 µM) for 24, 48 or 72 h. Cell viability was assessed using the CCK-8 assay. **d** NOZ and SGC cells were exposed to 1 µM casticin for 24 h, 48 or 72 h. **f**, **g** Casticin suppressed colony formation of NOZ and SGC996 cells. Cells were exposed to casticin (0, 1, 4, 7 µM) and were allowed to form colonies for 14 days. All data are presented as the means ± standard deviations, and each experiment was repeated 3 times. Significant differences compared with the control are indicated by *p < 0.05, **p < 0.01, and ***p < 0.001

Casticin was obtained from Sigma-Aldrich (St. Louis, MO, USA) (Fig. 1a), dissolved in dimethyl sulfoxide (DMSO), and stored at −20 °C. The final DMSO concentration used was less than 0.1%. A cell counting kit-8 (CCK-8), Hoechst 33342, and Rhodamine 123 were purchased from Sigma-Aldrich. Pan-caspase inhibitor (Z-VAD-FMK) and PI3K inhibitor (LY294002) were obtained from Abcam (Cambridge, MA, USA). An annexin V/propidium iodide (PI) apoptosis kit was purchased from Invitrogen (Carlsbad, CA, USA). TUNEL Apoptosis Assay Kit was purchased from Beyotime (Shanghai, China). All antibodies were purchased from Santa Cruz Biotechnology (Santa Cruz, CA, USA). All cell culture supplies were obtained from Invitrogen Gibco (Carlsbad, CA, USA).

### Cell culture

The human GBC cell lines NOZ and SGC996 were purchased from the Cell Bank of the Type Culture Collection of the Chinese Academy of Sciences (Shanghai, China). NOZ cells were cultured in William’s medium, and SGC996 cells were cultured in 1640 medium. All media were supplemented with 100 µg/ml streptomycin and 100 U/ml penicillin (Hyclone, Logan, UT, USA) and 10% fetal bovine serum (FBS, Gibco). The cells were cultured at 37 °C in a humidified incubator with 5% CO_2_.

### Cell viability assay

The viability of GBC cells treated with casticin was evaluated using a CCK-8 assay. Cells were seeded into 96-well plates at a density of 4000 cells/well and were cultured for 16–24 h. The cells were subsequently treated with various concentrations of casticin (0, 0.1, 0.5, 1, 4, 7, 10 µM) for 24, 48 or 72 h. After the treatment, CCK-8 (10 µl) was added to each well, and the cells were incubated for 3 h away from light. Absorbance was measured at 450 nm using a microplate reader (Bio-Tek, Norcross, GA, USA). Cell viability was calculated using the following formula: cell viability = (OD of control − OD of treatment)/(OD of control − OD of blank) * 100%. The assay was repeated 3 times.

### Colony formation assay

The SGC996 and NOZ cells were seeded into 12-well plates with casticin (0, 1, 4, 7 µM) for 15 days. Then, the cells were fixed with 10% formalin and stained with 0.1% crystal violet (Sigma-Aldrich). After washing, the plates were dried up and the colonies (with more than 50 cells) were observed under a microscope (Leica, Wetzlar, Germany).

### Cell cycle analyses

SGC996 and NOZ cells were treated with casticin (0, 1, 4, 7 µM) for 48 h. The cells were subsequently collected, washed with phosphate-buffered saline (PBS), and fixed with 75% ethanol overnight. The cells were then centrifuged (1500 rpm, 5 min), incubated with 10 mg⁄ml RNase and 1 mg/ml PI at 37 °C for 30 min away from light. Ultimately, cell cycle distribution was analyzed by flow cytometry (FACSCalibur BD, Bedford, MA, USA).

### Annexin V/PI staining assay for apoptosis

SGC996 and NOZ cells were treated with casticin (0, 1, 4, 7 µM) for 48 h. Then, the cells were collected and washed with PBS. After centrifugation (1500 rpm, 5 min), the cells were combined with 1× Annexin V binding buffer and then incubated with 5 µl Annexin V and PI at 37 °C for 30 min. Cell apoptosis was measured using flow cytometry.

### Hoechst 33342 staining

SGC996 and NOZ cells were treated with casticin (0, 1, 4, 7 µM) for 48 h. The cells were subsequently fixed with 1 ml methanol/acetic acid (3:1) for 20 min. The fixed GBC cells were washed with PBS and stained with 5 µg/ml Hoechst 33342 for 15 min at 37 °C. A fluorescence microscope (Leica, Wetzlar, Germany) was used to observe the morphological changes.

### TUNEL assay

TUNEL assay was performed on GBC cells and paraffin-embedded tissue sections using the one-step TUNEL apoptosis assay kit (Beyotime, Shanghai, China) according to the manufacturer’s instructions. After casticin treatment, samples were incubated with TUNEL reaction mixture for 1 h at 37 °C in the dark and then washed twice in PBS. The condensed or fragmented nuclei of apoptotic cells were observed using fluorescence microscopy at 400× magnification.

### Mitochondrial membrane potential (ΔΨm) assay

The cells were treated with casticin (0, 1, 4, 7 µM) for 48 h, collected and washed with cold PBS. Then, the samples were incubated with Rhodamine 123 in a 5% CO2 incubator at 37 °C for 20 min in the dark. Finally, the cells were analyzed by flow cytometry.

### Western blot analysis

Western blot analysis was used to evaluate the changes in protein expression, as previously described [14]. Proteins were separated using 10% sodium dodecyl sulfate polyacrylamide gel electrophoresis (SDS-PAGE) and were then transferred to polyvinylidene difluoride (PVDF) membranes. The membranes were blocked for 1 h at 37 °C and incubated with primary antibodies against Bcl-2, Bax, cleaved caspase-9, cleaved caspase-3, cleaved PARP, CDK4, protein kinase B (AKT), p-AKT, p27, cyclinD1 and GAPDH overnight at 4 °C. Next, the membranes were incubated with secondary antibodies for 1 h at 37 °C. Proteins were observed using a Gel Doc 2000 (Bio-Rad, USA).

### In vivo tumor xenograft study

Male nude mice (aged 4–6 weeks, weighted 18–22 g) were purchased from Shanghai SLAC Laboratory Animal Co Ltd (Shanghai, People’s Republic of China). The animals were housed at 25 ± 2 °C at a relative humidity of 70 ± 5% under natural light/dark conditions for 1 week and were allowed free access to food and water. NOZ cells (at a density of 1 × 10^6^ cells in 0.2 ml) were injected into the right axilla of each mouse. Twenty-four hours later, the mice were randomly divided into 3 groups (control, 10, 20 mg/kg; 7 mice per group). Mice in control group were intraperitoneally injected with (10% DMSO + 90% PBS). The other groups received administered casticin (10 or 20 mg/kg) every 2 days for up to 25 days. On day 26, the animals were sacrificed, and the tumors were dissected and weighed. The animal experiments were performed in strict accordance with international ethical guidelines and the National Institutes of Health Guide for the Care and Use of Laboratory Animals (SYXK [Shanghai] 2013-0106).

### Immunohistochemistry and HE staining

After the mice were sacrificed, tumors from the nude mice treated with different concentrations of casticin were resected and immediately fixed in 10% formalin, embedded in paraffin, cut into 5 mm sections and mounted on slides. The expression patterns of Ki67, cyclinD1 and p-Akt were analyzed using immunohistochemical streptavidin-peroxidase staining (IHC) and hematoxylin and eosin (HE) staining was used for histopathological examination.

### Statistical analysis

All experiments were performed at least 3 times, and the results are expressed as the means ± standard deviations unless otherwise stated. The Student’s t test was used to compare the differences between the treated groups and the corresponding control groups. p < 0.05 was considered statistically significant.

## Results and discussion

### Casticin inhibits the proliferation and viability of NOZ and SGC996 cells

Cell proliferation was evaluated using the CCK-8 assay, which demonstrated that NOZ and SGC996 cell proliferation rates were significantly inhibited by casticin in a dose- and time-dependent manner (Fig. 1b–d). However, casticin treatment did not significantly inhibit 293T viability (Fig. 1e). The inhibition of casticin-treated GBC cells was moderate at 48 h, and the IC50 of NOZ and SGC996 cells was approximately 2 µM at 48 h. Therefore, we selected 1, 4 and 7 µM as the concentrations to use in subsequent experiments. The colony formation assay was used to detect the proliferation of single cell. The NOZ and SGC996 cells were treated with at various concentrations (0, 1, 4, 7 µM) for about 2 weeks. As shown in Fig. 1f, g, the number and size of colonies derived from casticin-treated cells were markedly smaller compared with the control group. These data demonstrate that casticin can inhibit the proliferation and viability of GBC cells.

### Casticin induces mitochondrial-dependent apoptosis in NOZ and SGC996 cells

**Fig. 2:**
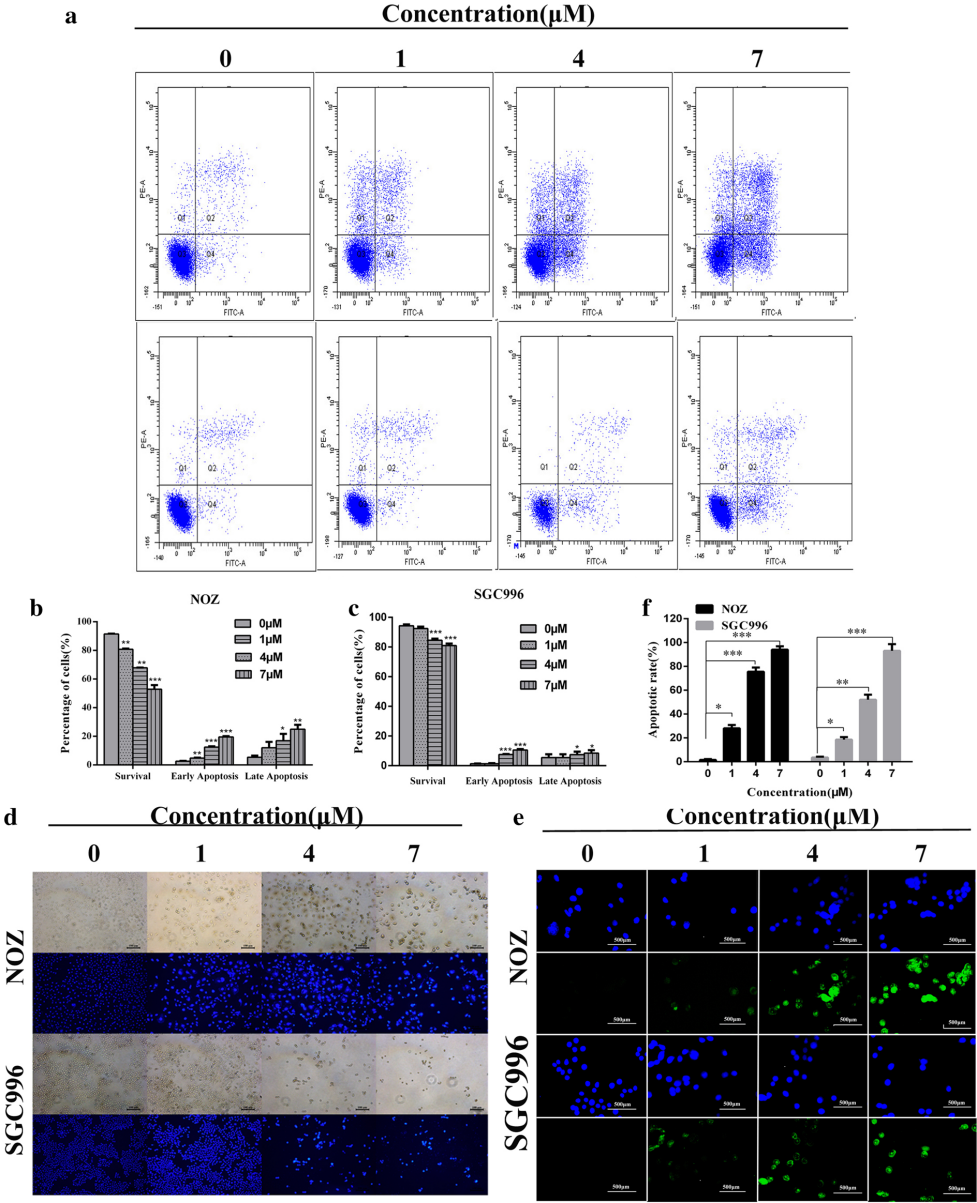
Casticin induces mitochondrial-dependent apoptosis in NOZ and SGC996 cells. **a**–**c** NOZ and SGC996 cells were treated with casticin (0, 1, 4, 7 µM) for 48 h. The Q3 quadrant (Annexin V−/PI−), Q4 quadrant (Annexin V+/PI−), and Q2 quadrant (Annexin V+/PI+) indicate the percentages of normal cells, cells in early apoptosis, and cells in late apoptosis, respectively. **d** NOZ and SGC996 cells were exposed to casticin for 48 h, and nuclear morphological changes associated with apoptosis were evaluated by Hoechst 33342 staining. **d**–**f** Representative images of TUNEL assay of different casticin-treatment in GBC cells. All data are presented as the means ± standard deviations and each experiment was repeated 3 times. Significant differences compared with the control are indicated by *p < 0.05, **p < 0.01, and ***p < 0.001

We investigated the effect of casticin on apoptosis in GBC cells using flow cytometry and Hoechst 33342 staining. Compared with the control group, the percentages of casticin-treated cells in the early and late apoptosis stages were strikingly elevated in a dose-dependent manner (Fig. 2a–c). In a subsequent experiment, we treated NOZ and SGC996 cells with various concentrations (0, 1, 4, 7 µM) for 48 h, and stained the cells with Hoechst 33342. As shown in Fig. 2d, the casticin-treated cells exhibited markedly increased chromatin condensation and fragmentation compared with the control group, in which cells were round and homogeneously stained. The result was consistent with the flow cytometry data, and together, these data indicate that casticin can induce apoptosis in NOZ and SGC996 cells. TUNEL analysis also showed more apoptotic cells in casticin-treatment GBC cells (Fig. 2e).

**Fig. 3:**
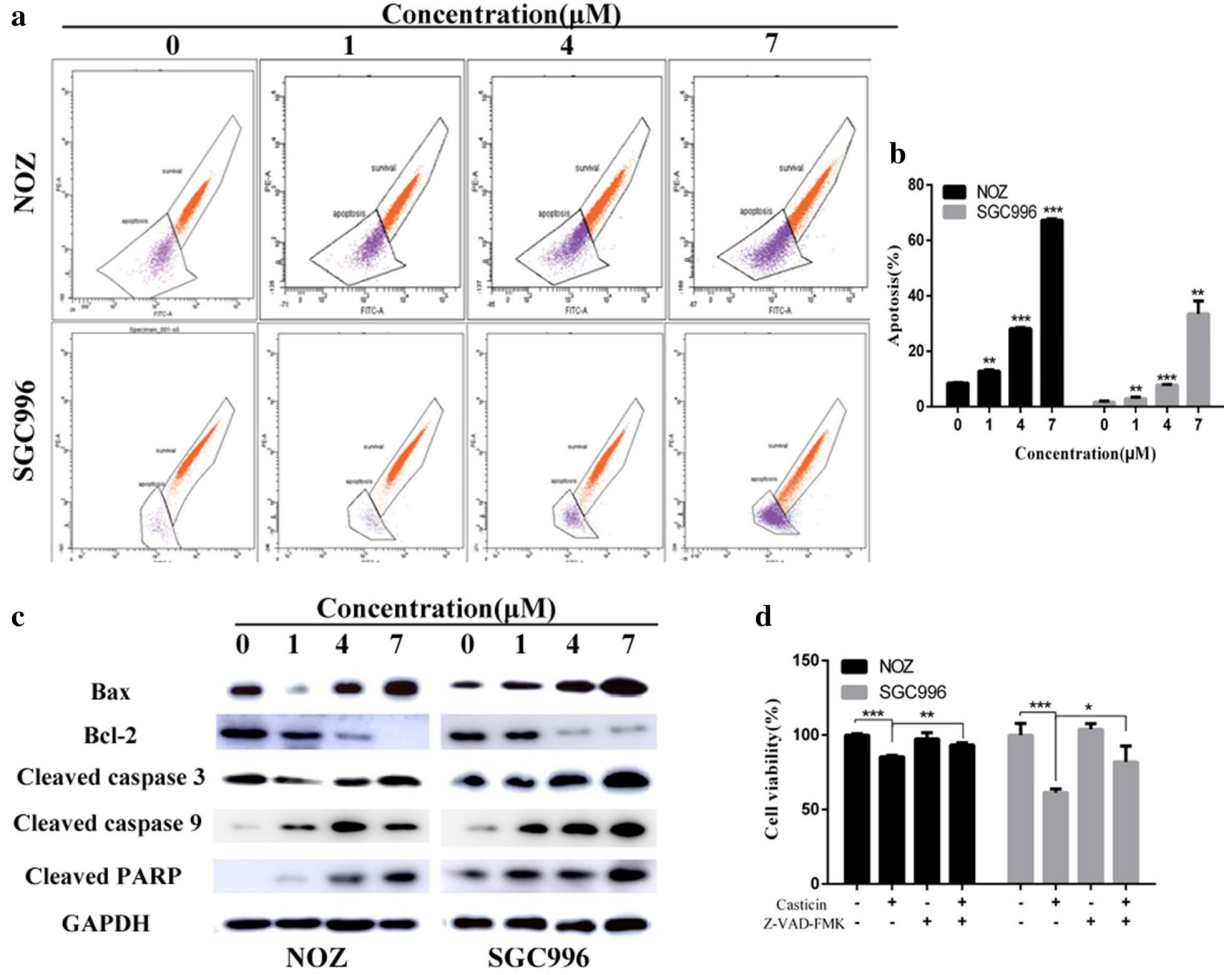
Casticin induces mitochondrial-dependent apoptosis in NOZ and SGC996 cells. **a**, **b** Flow cytometry analysis of the mitochondrial membrane potential (ΔΨm). NOZ and SGC996 cells were treated with casticin (0, 1, 4, 7 µM) and stained with Rhodamine 123. Cells with high ΔΨm are marked “survival”, and those with low ΔΨm are marked “apoptosis”. The percentages of cells with low ΔΨm (apoptosis) are shown. **c** Apoptosis-related proteins in NOZ and SGC996 cells were analyzed by western blot. GAPDH was used as a loading control. **d** After pretreatment with 5 mM Z-VAD-FMK for 1 h, GBC cells were incubated with 4 µM casticin for 24 h, and cellular viability was determined. All data are presented as the means ± standard deviations. Significant differences compared with the control were indicated by *p < 0.05, **p < 0.01, and ***p < 0.001

Mitochondrial damage to cells results in perturbation of the mitochondrial membrane potential (ΔΨm) [15]. We evaluated changes in the ΔΨm in NOZ and SGC996 cells using Rhodamine 123 staining, as the decrease in the intensity of Rhodamine 123 staining reflects mitochondrial membrane potential and integrity. As shown in Fig. 3a, b, ΔΨm decreased in a dose-dependent manner, indicating that casticin induces mitochondrial-dependent apoptosis.

**Fig. 4:**
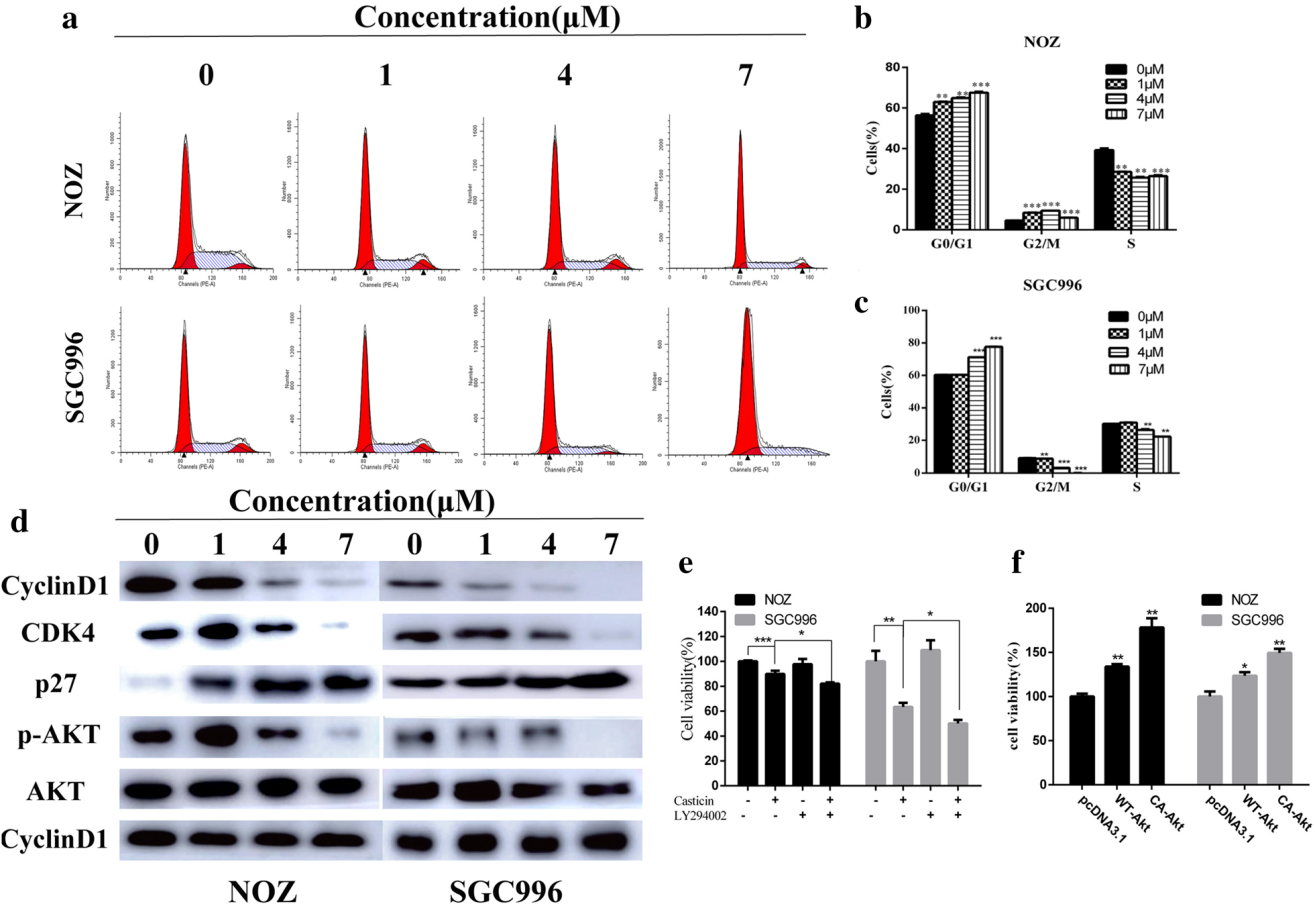
Casticin induces G0/G1 arrest by inactiving the AKT pathway. **a**–**c** NOZ and SGC996 cells were treated with casticin (0, 1, 4, 7 µM) for 48 h. The cell cycle distribution was analyzed by flow cytometry. **d** Expression levels of cyclinD1, CDK4, p27, p-AKT and AKT were measured using western blot analysis. **e** After pretreatment with 50 µM LY294002 (Akt inhibitor) for 1 h, GBC cells were incubated with 4 µM casticin for 24 h, and cellular viability was determined. **f** Cellular viability was determined after treatment with 4 µM casticin in cells transfected with WT-Akt, CA-Akt, or vehicle plasmid. All data are presented as the means ± standard deviations. Significant differences compared with the control were indicated by *p < 0.05, **p < 0.01, and ***p < 0.001

Caspases play critical roles in apoptosis initiation and maintenance [16, 17]. We explored the potential mechanism of casticin-induced apoptosis using western blot analysis. As shown in Figs. 3c and 4d, cleaved caspase-3, -9, -PARP, Bax and p27 were upregulated following exposure to casticin in a dose-dependent manner, whereas, Bcl-2, p-Akt and Bcl-2/Bax level significantly decreased compared with the control group. To confirm the results, we evaluated cell viability after treatment with casticin in the presence or absence of Z-VAD-FMK, a caspase inhibitor. As shown in Fig. 3d, Z-VAD-FMK can abolish casticin cytotoxicity in GBC cells. Together, these results indicate that casticin induces mitochondrial-dependent apoptosis in NOZ and SGC996 cells.

### Casticin induces G0/G1 arrest and inhibits proliferation regulated by an inactive AKT pathway

To determine whether casticin influences cell cycle progression, we investigated cell cycle distribution by flow cytometry. The results indicated that the proportions of G0/G1 cells increased in a dose-dependent manner in NOZ and SGC996 cells, indicating that casticin induced G0/G1 arrest (Fig. 4a–c). To further investigate the effect of casticin on cell cycle progression, we examined cycle-related protein expression using western blot analysis. Casticin treatment resulted in decreased levels of cyclinD1 and CDK4, consistent with a G0/G1 cell cycle arrest (Fig. 4d). A recent study identified p27 as an important cyclin-dependent kinase inhibitor that inhibits the activation of cyclin D-CDK4 complexes and induces cell cycle arrest at the G0/G1 and G2/M phases [18]. In addition, Akt pathway regulates cell cycle progression and cell proliferation by influencing p27, and the results presented in Fig. 4d was consistent with these findings.

To determine if casticin-induced proliferation inhibition was regulated by inhibiting of Akt activity, we evaluated cell viability after treatment with casticin in the presence or absence of LY294002, a PI3K/Akt inhibitor. As shown in Fig. 4e, LY294002 enhanced GBC cells death. To further confirm the results, we transiently transfected wild-type Akt and constitutively active Akt into GBC cells, and then we treated with casticin and evaluated the viability. As shown in Fig. 4f, overexpression of wild-type Akt and constitutively active Akt can abolish casticin cytotoxicity in GBC cells.

Therefore, we conclude that casticin induces G0/G1 arrest via Akt signaling pathway, and that the modulation of Akt signaling also accounts for the anti-proliferative effect of casticin.

### Casticin inhibits tumor growth in vivo

**Fig. 5:**
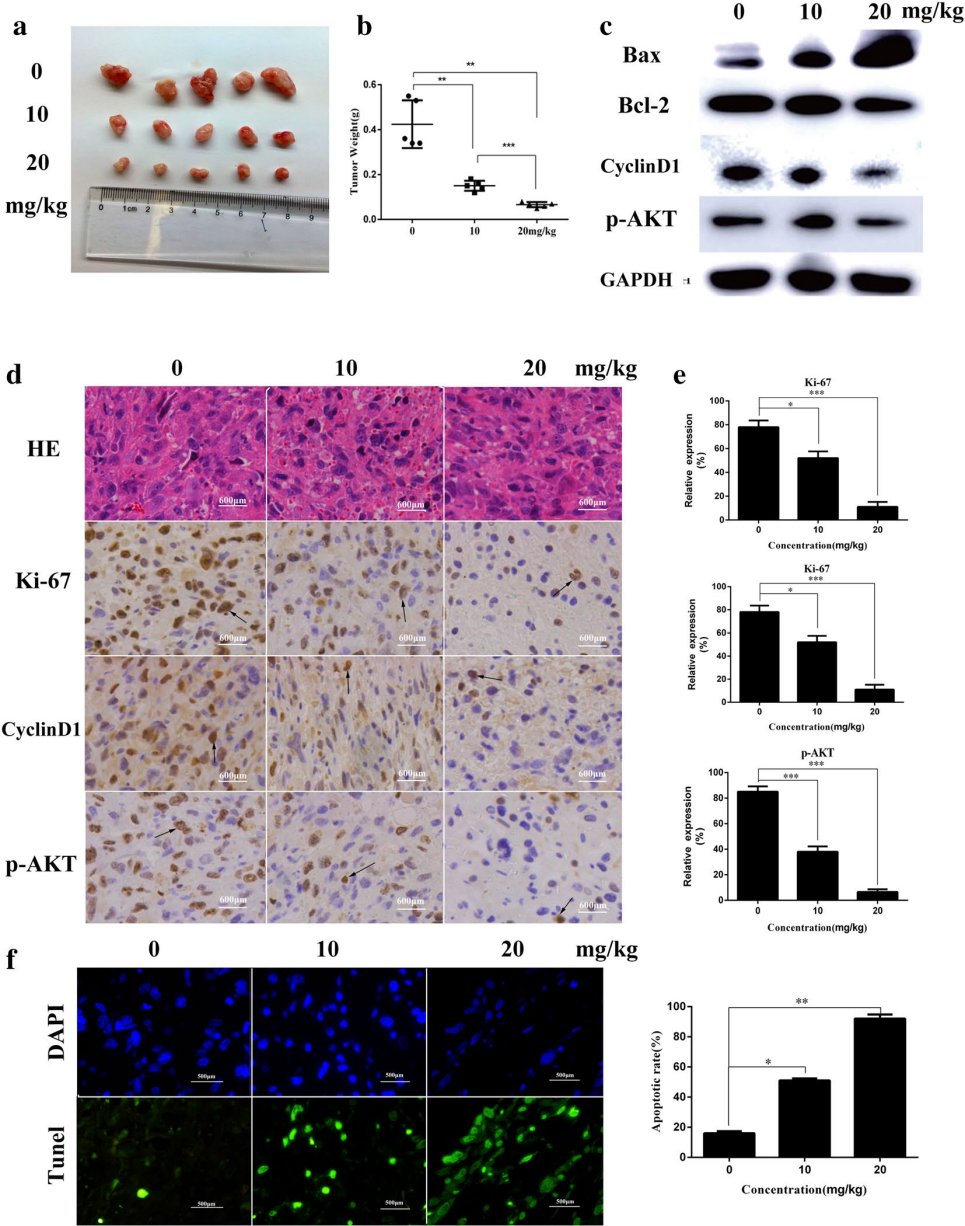
Casticin inhibits tumor growth in vivo. **a** Different concentrations (10% DMSO + 90% PBS, 10 and 20 mg/kg) casticin were injected into nude mice after inoculated NOZ cells every 2 days. Images of 5 representative mice (n = 7) from each group are presented to show the sizes of the resulting tumors. **b** Tumors were excised from the animals and weighed. **c**–**e** Bcl-2, cyclinD1, p-AKT and Ki-67 expression levels were analyzed using HE, IHC staining and western blot analysis. **f** Representative images of TUNEL assay of tumor xenografts (original magnifications: ×400). All data are presented as the means ± standard deviations. Significant differences from the control were indicated by *p < 0.05, **p < 0.01, and ***p < 0.001

To evaluate the anti-cancer effect of casticin in vivo, we injected mice with 10% DMSO + 90% PBS (control group) or casticin at a concentration of either 10 or 20 mg/kg every 2 days after their inoculation with NOZ cells. We found that casticin inhibits tumor growth in a dose-dependent manner (Fig. 5a, b). Based on this observation, we performed western blot analysis, HE and IHC analysis. As shown in Fig. 5c–e, Bcl-2, cyclinD1, p-AKT and ki-67 expression levels were strikingly reduced, and Bax expression level was significantly elevated in casticin-treated groups compared with the control group. Moreover, tunel analysis showed more apoptotic cells in casticin-treated groups compared with the control group (Fig. 5f). These results are consist with the in vitro effects of casticin.

GBC is the most common and fatal cancer in biliary system, and surgical resection is the only effective treatment option. Thus, it is essential to identify novel effective treatments for GBC. Traditional Chinese medicine has been extensively used to treat various diseases for thousands of years. As an active compound isolated from Vitex Fructus, casticin can inhibit proinflammatory cytokines and inflammatory mediators, such as NO and PGE2, by blocking the activation of NF-ΚB, Akt, and MAPK signaling [19]. Recent studies have shown that casticin inhibits proliferation and induces apoptosis in various cancer cells in vitro. However, there have been no in vivo tumor xenograft studies evaluating the anti-cancer effect of casticin. In this study, we investigated NOZ and SGC996 cell proliferation and viability using CCK-8 analysis and colony formation assays. We found that casticin can inhibit NOZ and SGC996 cell proliferation, and casticin cannot significantly inhibit 293T cell viability. Therefore, we propose that casticin represents a new and promising therapeutic agent for gallbladder cancer. Moreover, we evaluated the effect of casticin treatment in mice with xenografted tumors. Based on the weight and volume of the tumors, we conclude that casticin exerts anti-cancer activity in GBC in vivo. In addition, the expression of related proteins expression using western blot analysis, HE and IHC staining, the results of which were in accordance with our in vitro assays.

We also evaluated the effect of casticin on apoptosis using flow cytometry, Hoechst 33342 staining and tunel analysis. Apoptosis is generally characterized as specific morphological changes, such as cell shrinkage, nuclear or cytoplasmic fragmentation, chromatin condensation and the formation of dense bodies that are phagocytosed by neighboring cells [20]. As shown in Fig. 2d, NOZ and SGC996 cells treated with casticin exhibited markedly increased chromatin condensation and fragmentation compared with the control group cells, which were round and homogeneously stained. In addition, the proportions of cells in early and late apoptosis stages in the casticin-treated groups were strikingly elevated in a dose-dependent manner.

Various mechanisms have been suggested to contribute to the progression of gallbladder cancer, in particular mutations in components of cell cycle or apoptotic pathways, and the processes of signal transduction, angiogenesis, invasion, and metastasis [21–23]. Apoptosis signaling cascades can be divided into 2 major pathways: a death-receptor-induced extrinsic pathway and a mitochondria-apoptosome-mediated intrinsic pathway [24]. In this study, we found that mitochondrial-dependent apoptosis was involved in casticin induced apoptosis. Bax and Bcl-2 are important regulators of the mitochondria-mediated apoptosis pathway, and the balance of these 2 factors is crucial for cell survival and cell death. The antiapoptotic factor Bcl-2 has been shown to prevent apoptosis by forming a heterodimer with proapoptotic factors, such as Bax, resulting in proapoptotic effects [25]. Activation of Bcl-2 family proteins can induce the mitochondrial permeabilization, and can induce caspase-9 activation, which subsequently induces the cleavage of procaspase-3 [26]. Caspase-3 is a key executioner caspase that it is capable of cleaving many important cellular substrates, including PARP [27]. In this study, we demonstrated that Bax expression significantly increased and that Bcl-2 expression decreased in response to casticin treatment both in vitro and in vivo. In addition, casticin significantly enhanced the enzymatic activity levels of caspase-3, caspase-9 and PARP (Fig. 3c). Moreover, ΔΨm decreased in a dose-dependent manner after 48 h of incubation with casticin. Together, these results suggest casticin induced apoptosis occurs through the mitochondria-dependent pathway.

The induction of cell cycle arrest at a specific checkpoint and thereby inducing apoptosis is a common mechanism for the cytotoxic effects of anticancer drugs [28]. In this study, we investigated cycle distribution using flow cytometry. The data showed that the proportion of G0/G1 cells increased in a dose-dependent manner, indicating that casticin can induce G0/G1 arrest. Cell cycle progression is highly regulated by a series of cell cycle checkpoint proteins, such as the cyclins and CDKs. Among these proteins, cyclinD and E, together with CDK2, CDK4, or CDK6, play major roles in DNA replication and mitosis by regulating G0/G1 phase of the cell cycle [29]. Therefore, we investigated the expression of cylinD1 and CDK4 in casticin-treated GBC cells and found that cyclinD1 and CDK4 contributed to G0/G1 arrest.

The PI3K/AKT pathway is one of the major signaling pathways involved in the progression of various tumors and is associated with cancer progression and invasion [30]. AKT is a key downstream effector of PI3K and is down-regulated in various cancers, including osteosarcoma and prostate cancer [31]. Previous studies have demonstrated that Akt inactivation might inhibit the expression of proteins associated with events that mediate the cancer development and progression, including apoptosis and cell cycle progression [29, 32, 33]. In our study, the results of the western blot analysis demonstrated that casticin significantly decreased p-Akt expression and that this effect was accompanied by an increase in p27. Inactivation of Akt leads to the increased expression of p27, and decreased expression of cyclinD1/CDK4 decreased, which contributed to G0/G1 arrest. Furthermore, Akt inactivation can lead to upregulation of Bad expression and the downregulation of Bcl-2 expression, which associated cell apoptosis. In summary, we suggest that the Akt signaling pathway is involved in casticin-induced cell apoptosis and cell cycle arrest.

## Conclusions

Taken together, these findings indicate that the Akt signaling pathway is involved in casticin-induced cell apoptosis and cycle arrest. Furthermore, the intrinsic mitochondrial pathway is involved in casticin-induced apoptosis. Therefore, we suggest that casticin might be a novel and effective therapy for GBC.

## Abbreviations

PARP: poly ADP-ribose polymerase
CDK4: cyclin-dependent kinase4
Akt: protein kinase B
p-Akt: phosphorylated protein kinase B
DMSO: dimethyl sulfoxide
CCK-8: cell counting kit-8
Z-VAD-FMK: pan-caspase inhibitor
FITC: fluorescein isothiocyanate
PI: propidium iodide
IHC: immunohistochemical streptavidin-peroxidase staining
HE: hematoxylin and eosin
LY294002: 2-(4-morpholinyl)-8-phenyl-4H-1-benzopyran-4-one
PBS: phosphate buffered saline
PI3 K: phosphatidylinositol 3-kinase
z-VAD-fmk: Z-Val-Ala-Asp(OMe)-CH2F
GAPDH: glyceraldehyde 3-phosphate dehydrogenase
